# MicroRNAs at the Interface between Osteogenesis and Angiogenesis as Targets for Bone Regeneration

**DOI:** 10.3390/cells8020121

**Published:** 2019-02-03

**Authors:** Leopold F. Fröhlich

**Affiliations:** Department of Cranio-Maxillofacial Surgery, University of Münster, Albert-Schweitzer-Campus 1, 48149 Münster, Germany; leopold.froehlich@ukmuenster.de; Tel.: +49-251-834-7007

**Keywords:** bone angiogenesis, osteogenesis, angiogenic-osteogenic coupling, microRNAs, bone regeneration, bone formation, bone tissue-engineering, angiomiRs, osteomiRs, hypoxamiRs

## Abstract

Bone formation and regeneration is a multistep complex process crucially determined by the formation of blood vessels in the growth plate region. This is preceded by the expression of growth factors, notably the vascular endothelial growth factor (VEGF), secreted by osteogenic cells, as well as the corresponding response of endothelial cells, although the exact mechanisms remain to be clarified. Thereby, coordinated coupling between osteogenesis and angiogenesis is initiated and sustained. The precise interplay of these two fundamental processes is crucial during times of rapid bone growth or fracture repair in adults. Deviations in this balance might lead to pathologic conditions such as osteoarthritis and ectopic bone formation. Besides VEGF, the recently discovered important regulatory and modifying functions of microRNAs also support this key mechanism. These comprise two principal categories of microRNAs that were identified with specific functions in bone formation (osteomiRs) and/or angiogenesis (angiomiRs). However, as hypoxia is a major driving force behind bone angiogenesis, a third group involved in this process is represented by hypoxia-inducible microRNAs (hypoxamiRs). This review was focused on the identification of microRNAs that were found to have an active role in osteogenesis as well as angiogenesis to date that were termed “CouplingmiRs (CPLGmiRs)”. Outlined representatives therefore represent microRNAs that already have been associated with an active role in osteogenic-angiogenic coupling or are presumed to have its potential. Elucidation of the molecular mechanisms governing bone angiogenesis are of great relevance for improving therapeutic options in bone regeneration, tissue-engineering, and the treatment of bone-related diseases.

## 1. Introduction

The replacement of large bone defects and the availability of adequate tissue-engineered bone remains a major clinical challenge. This tremendous demand results from the high incidence of large segmental bone defects due to trauma, congenital malformations, ageing, or bone-related diseases such as osteoporosis, inflammation or tumors [1]. However, the development of gene therapy approaches in recent years demonstrated that tissue-engineered bone offers new therapeutic strategies to repair tissue defects. Thereby, one of the major disadvantages in the clinical use of engineered bone constructs so far, i.e., the inability to provide sufficient blood supply in the initial phase after implantation which leads to insufficient cell integration and cell death, could be overcome [2]. As an explanation for this shortcoming, the function of angiogenesis—the process of forming new blood vessels from pre-existing vasculature—in bone regeneration is still poorly defined, and the molecular mechanisms that regulate angiogenesis in bone are only just starting to be unraveled. Angiogenesis, a term that was coined in 1935 to describe the formation of blood vessels in the placenta, occurs during normal vertebrate embryogenesis but also as a response to pathophysiological circumstances during the processes of tumor formation, wound healing and tissue regeneration [3,4]. Elucidating the wide orchestrating variety of signal pathways and stimuli linking angiogenesis and bone formation on the molecular level is therefore of great interest. In this context, the gained information will yield improved bone replacement in fracture healing, or prevention of bone loss in osteoporosis and therapeutically-induced reparative angiogenesis.

Bone, with its main function in supporting and withstanding mechanical forces, is a mineralized mesenchymal tissue that also possesses an important role in maintaining mineral homeostasis and the energy metabolism of the organism [5]. The formation of bone, which is either developed by the endochondral or by the intramembranous ossification program, is a complex process which depends on physiological interaction with blood vessels that are simultaneously formed [6,7,8,9]. Endochondral ossification designates the process of long bone formation which results from the intermediate generation of a primordial cartilage skeleton composed of mesenchymal stem cells (MSCs) that differentiate into chondrocytes and only at a later stage gradually transform into mature bone recruited by different types of bone-forming cells [6]. Intramembranous ossification, in contrast, denominates the process of establishing flat bones where condensed MSCs directly differentiate into osteoblasts, forming an ossification center. This pathway leads to the formation of craniofacial, calvarial, and clavicle bones [10]. In either type of bone development, angiogenesis—the invasion of small blood vessels derived from preexisting blood vessels—is required [11,12,13]. Bone angiogenesis is induced by growth factors expressed by osteogenic cells such as hypertrophic chondrocytes and osteoblasts at an early stage during osteogenesis [14]. Thereby, the transport of oxygen and nutrients, as well as the further recruitment of osteoprogenitor cells (MSCs) and osteoblasts, are facilitated. In the later phase of bone formation, angiogenesis is essential for trabecular (spongy, cancellous) bone formation and for maturation of the newly-formed bone by close coordination of mineralization and vascularization in either type of bone. In addition, endochondral angiogenesis is particularly important for the replacement of cartilaginous structures at the primary ossification center which generates the bone marrow cavity, and, at a later stage, in establishing the secondary ossification center at the epiphyses (the distal end of long bones) [15,16]. Recent in vitro experiments in transgenic mice demonstrated that this task is accomplished by a specialized type of blood vessel, i.e., the so-called H-type that is present in long bones [17]. This underlines the fact that, comparable to other organs, the acquisition and maintenance of specialized properties by endothelial cells (ECs) is very important for the functional homeostasis in bone.

Bone is a tissue that has to undergo permanent remodeling and requires a counterbalanced process between the anabolic activities of bone formation (osteogenic) cells and the catabolic activities of bone resorption (osteoclast) cells. This activity enforces continuous self-renewal of bone, thereby maintaining an appropriate bone mass and calcium equilibrium. Interference with bone homeostasis could prevent tissue formation, leading to immature or abnormal bone formation [18]. Impaired blood vessel formation, as found in age-related or disease-induced bone loss, therefore, could also result in imbalanced or defective bone formation [19]. In support of this, a reduced density of blood vessels altering the microcirculation was found to be present in osteoporotic bone in mice and humans, that may lead to local abnormal bone metabolism and provoke an increased risk of fracturing [20,21]. Furthermore, the genetic program of bone angiogenesis needs reactivation during callus formation in fracture healing, which represents a complex process that has not yet been fully elucidated [22].

## 2. Molecular Regulation of Bone Angiogenesis

Bone angiogenesis is mainly governed by a spectrum of transcription factors and growth factors which have been mostly elaborated for endochondral ossification so far [23,24]. It involves interactive signaling between cells of the skeletal system, namely chondrocytes and osteoblasts, and cells derived from the bone vascular system, primarily ECs. Initially, the formation of blood vessels is promoted by osteogenic cells producing pro-angiogenic factors which, in turn, later support the settlement of osteoprogenitor cells [25]. One of the major driving forces behind angiogenic-osteogenic coupling in bone, where oxygen concentrations below 1% are encountered, is the necessity of supplying the tissue with oxygen [15,26,27]. Via tightly hypoxia-regulated induction of the transcriptional activator hypoxia inducible factor (HIF), a cascade of target genes which are involved in a wide variety of biological processes including energy metabolism, erythropoiesis, cell survival, apoptosis and angiogenesis including the major angiogenic regulator vascular endothelial growth factor (VEGF) are expressed in osteogenic cells [28]. The direct mediator of oxygen-dependent modifications of the HIF factors is the von Hippel-Lindau tumor suppressor protein (pVHL), an E3 ubiquitin ligase that targets HIF-1a for proteasomal degradation. During endochondral ossification, VEGF-A is produced by both chondrocytes, particularly in a later stage in which they undergo hypertrophy, and by osteoblasts [29]. As proof of principle, it has been demonstrated that in mice which produce only altered expression levels of a soluble form of VEGF (VEGF120), delayed blood vessel invasion into the primary ossification center and altered osteoblast differentiation in vitro occurs [11]. In addition to HIFs also the fibroblast growth factor (FGF) family of signaling molecules with the members FGF2 and FGF9, and their receptors FGFR1 and 2, were found to be involved in the transcriptional regulation of VEGFA and VEGFR2 expression during formation of blood vessels in bone [30]. Recently, Notch signaling has been implicated as a response to VEGF signaling in ECs in osteogenic-angiogenic coupling [31]. While in other tissues NOTCH expression generally negatively regulates angiogenesis, it seems to have the opposite role in enchondral bone formation by promoting EC proliferation and vessel growth. In a reciprocal fashion, ECs respond by an angiocrine release of NOGGIN, an antagonist of the bone morphogenetic protein (BMP) pathway that stimulates the maturation of hypertrophic chondrocytes expressing VEGF in the growth plate. This could be nicely demonstrated in mice lacking Notch specifically in ECs, which demonstrated reduced bone angiogenesis due to a loss of type-H blood vessels, as well as mutant bone formation and VEGF expression. Other major players are represented by members of matrix metalloproteases (MMPs) due to their proteolytic activity originating from osteoclasts and vascular cells [32]. MMPs also seem to mediate intracellular signaling involving extracellular matrix-integrin interactions necessary during bone angiogenesis and bone remodeling. Besides VEGFA, the placental growth factor (PlGF/PGF), a member of the VEGF family which binds to VEGFR1, has been found to play an exclusively important role in callus remodeling during fracture healing [33,34]. Other known modulating factors of angiogenesis during bone repair include transforming growth factor beta (TGF-β), BMPs, and growth differentiation factor (GDF) [35]. 

## 3. The Role of MicroRNAs 

MicroRNAs (miRs/miRNAs) are a newly discovered expanding class of endogenous small, non-coding RNAs that positively or negatively regulate gene expression and cellular processes via the RNA interference pathway [5,36,37,38,39,40,41,42,43,44]. By targeting messenger RNA transcripts post-translationally, they provoke either translational repression or degradation, depending on the degree of sequence homology. Upon precursor transcription from intronic or polycistronic genomic loci by RNA polymerase II, biogenesis of the primary miRNA (pri-miRNA) transcript takes place by a two-step processing mechanism involving the RNAses Drosha and DICER (DGCR8 RNase III complex) [45,46,47]. Thereby, single or multiple miRNAs that form hairpin-like structures are exported to the cytoplasm by an exportin 5- and RAN-GTP-dependent process, and cleaved. However, in an alternative non-canonical pathway, miRNAs can be configured by direct transcription or refolded spliced introns as endo-shRNAs (endogenous short hairpin RNAs) or as mirtrons, respectively [48]. Subsequently, the targeting strand of the double-stranded mature miRNAs that are 18 to 22 nucleotides in length is integrated into the RNA-induced silencing complex (RISC) with the help of Argonaute proteins. RISC can finally bind specific target (or so-called “seed”) sequences of mRNAs represented by 2 to 8 nucleotides located mostly in their 3´-untranslated regions (UTRs) [49,50]. Up- or down- regulation of the miRNA itself by stage- and tissue-specific expression patterns during development can lead to modified expression of its target genes. Thus, miRNAs function as decisive regulatory molecules in many different cellular activities such as development, proliferation, and differentiation, metabolism, or apoptotic cell death, or even cell fate determination and maintenance (e.g., pluripotency control of embryonic stem cells) [51,52,53,54,55,56,57,58,59,60]. Moreover, relevant to bone biology, miRNAs have also been identified to acquire endocrine or paracrine functions by their secretion into the blood stream where they subsequently circulate [61]. Consistent with this finding, it was determined that a single miRNA can be involved in coordinating genetic networks by simultaneously regulating the endogenous expressions of multiple target genes. While miRNAs seem to function rather as auxiliary factors during normal physiological processes, their task seems to become more important under stress or disease-related conditions. Accordingly, disturbed miRNA expression is increasingly identified in a number of pathological conditions such as tumorigenesis or viral infection [53,62].

## 4. MicroRNAs in Bone Angiogenesis: OsteomiRs, AngiomiRs, and HypoxamiRs

It is now well established that miRNAs are physiologically relevant to all steps of bone as well as blood vessel formation during embryonic development and in maintenance during adulthood [63]. OsteomiRs have been identified to regulate chondrocyte, osteoblast, and osteoclast differentiation by positively targeting the principal osteogenic transcription factors and signaling molecules of osteogenesis [5,38,64,65,66,67,68,69]. In addition to regulating MSC commitment—i.e., the differentiation of precursor cells into chondrocytic and osteoblastic lineages—several studies showed that miRNAs also contribute to the maturation and function of these cells, suggesting also important roles in bone regeneration. Nevertheless, the exact mechanisms of skeletal miRNAs governing the complex interactions and signaling pathways of different bone-forming cells are only beginning to be elucidated. Deregulated miRNAs expression or even genetic variation by mutations or single nucleotide polymorphisms in miRNAs or their binding sites have been identified furthermore in bone disorders such as osteoporosis, osteosarcoma, osteopetrosis, osteogenesis imperfecta, osteoarthritis, and furthermore in bone fracture [63,70,71,72,73,74]. 

Increasing evidence further indicates that miRNAs act as pro- and anti-angiogenic regulators of adaptive blood vessel growth in normal cardiovascular development and in tumor angiogenesis [75,76,77,78,79,80]. The role of these so-called angiomiRs or vascular microRNAs in angiogenic development was initially discovered by the detection of severely disrupted blood vessel formation and delayed angiogenic capabilities of ECs in mid-gestational lethal mouse mutants for the miRNA precursor-processing enzyme Dicer [81]. These mutants die around embryonic day 12 to 14 of development due to vascular defects in the embryo and the yolk sac. Furthermore, a smooth muscle-specific *Dicer* deletion in the mouse exerted late embryonic lethality associated with extensive internal hemorrhage which could be explained by a significant loss of vascular contractile function, smooth muscle cell (SMC) differentiation, and vascular remodeling [82]. Knockdown experiments of *Dicer* in zebrafish moreover provoked a phenotype of pericardial edema and inadequate circulation. But also, loss-of-function of the EC-specific miR-126 in homozygous deficient mice caused defects in vascular integrity and angiogenesis [83]. These findings suggested that angiomiRs modulate crucial target genes in cells derived from angioblastic precursor cells and SMC, which are indispensable during embryonic angiogenesis. By investigating the function of Dicer in adult mice and human cells, considerable dysregulated angiogenesis related to growth factor release, ischemia, and wound healing could be revealed, reflecting important postnatal angiogenic functions [80,84,85]. To date, miRNA have been implicated in a long list of cardiovascular diseases comprising myocardial infarction, heart failure, stroke, peripheral and coronary artery disease and several more [86,87]. Nevertheless, the pathological implications of angiomiRs surfaced also with the help of endothelium-specific Dicer-deficient mice, as the ablation led to reduced tumor progression due to diminished angiogenesis, which is a prerequisite for tumor development [88]. For example, two miRNAs induced by VEGF expression (miRs-296, miRs-132) have been identified as candidates supporting the angiogenic switch during tumor formation i.e., the transition from a pre-vascular to a vascularized tumor phenotype [89,90]. In conclusion, the combination of Dicer-deficient angiogenic phenotypes suggests crucial roles for miRNAs in regulating structure and function of embryonic and postnatal blood vessel development. 

In the context of angiogenesis, an additional, very important category is a specialized subset of hypoxia-inducible miRNAs, whose increasing number of representatives was also termed hypoxamiRs [91,92,93,94,95,96]. Thus, reduced oxygen supply in ossification centers of bone stimulate the expression of VEGF and other angiogenic factors that lead to the development of blood vessel structures [97]. Additionally, hypoxia-regulated pathways have been attributed to regulatory functions such as smooth muscle cell proliferation and contractility, cardiac remodeling, cardiac metabolism and ischemic cardiovascular diseases [94]. Together with a variety of other target genes which are important for physiological low oxygen adaption, their expression is initiated by upregulation of the transcription factor hypoxia-inducible factor alpha (HIF) [98]. One group of hypoxamiRs are therefore upregulated following HIF expression (HIF-dependent hypoxamiRs), with the master hypoxamiR-210 being the most prominent example [99,100]. Hypoxia-dependently expressed miRNAs that affect HIF expression itself also belong to hypoxamiRs. Thus, for the adaptation to low oxygen conditions and induction of angiogenesis, HIF displays a unique role by controlling further upregulation of hypoxamiR-424 in ECs, which promotes its own protein stabilization [101]. A last group of hypoxamiRs, moreover, influences HIF expression in the absence of hypoxia. As an example, miR-31 decreases HIF-1α expression via the “factor-inhibiting HIF (FIH)” while the miR17-92 cluster suppresses HIF-1α upon c-MYC induction [102,103].

## 5. Specific MicroRNAs Implicated in Angiogenic-Osteogenic Coupling

**Figure 1:**
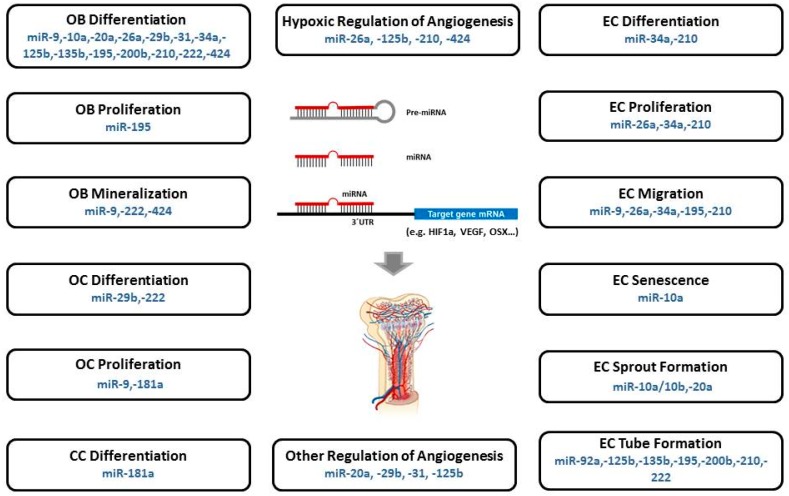
MicroRNAs (miRs/miRNAs) involved in the regulation and coupling of bone angiogenesis (“CouplingmiRs/CPLGmiRs”). Reported miRNAs contributing to the formation of blood vessels during the processes of formation, repair and regeneration of bone were allocated with the individual functions of their target genes during osteogenesis, angiogenesis, or hypoxic regulation of bone angiogenesis. OB, osteoblast; OC, osteoclast; CC, chondrocyte; EC, endothelial cell.

Taken together, the functions of osteomiRs, angiomiRs, and hypoxamiRs suggest the possibility that miRNAs will also have crucial roles in bone angiogenesis. Subsequently, miRNAs will be outlined that were found to have a significant function in osteogenesis as well as angiogenesis, and therefore represent miRNAs that have already been identified to have an active role in angiogenic-osteogenic coupling or are presumed to have its potential (Figure 1, Table 1). Collectively, these may also be referred to as “CouplingmiRs/CPLGmiRs”. MiRNAs with a confirmed function in this process could be employed as therapeutic targets in bone regeneration. Consequently, they could improve the coordination and enhancement of the endogenous osteogenesis and angiogenesis process. Elucidation of the molecular mechanisms governing osteoblast differentiation and angiogenesis are furthermore of great importance for improving the treatment of bone-related diseases.

### 5.1. MiR-9

miR-9 is a highly conserved microRNA, but exhibits a divergent expression pattern, and seems to modulate different targets in a cellular context- and developmental stage-specific manner [104]. The studies of Han et al. have demonstrated a regulatory role for miR-9 in the development and differentiation of human bone marrow derived MSCs (hBM-MSCs) and neural progenitor cells on proliferation, migration and differentiation [105]. In this regard, cell-autonomous effects have been described in vertebrates by effecting Notch, Wnt, and BAF53a expression. Furthermore, miR-9 has also been related to tissue repair processes involving human MSCs (hMSCs). Qu et al. provided evidence that increased miR-9 expression levels closely correlate with enhanced differentiation of MC3T3-E1 osteoblasts [106]. The effects of miR-9 on angiogenesis were studied by the same authors by using human umbilical vein endothelial cells (HUVECs). Here, miR-9 mimics transfection effectively increased VEGF, VE-cadherin, and FGF concentrations in the culture medium, leading to increased EC migration and capillary tube formation in vitro. The underlying molecular mechanism for both the regulation of osteoblast differentiation and angiogenesis was found in the activation of AMP-activated (AMPK) signaling. Conclusively, the results of Qu et al. imply a potential important role of miR-9 in regulating the process of bone injury repair, and therefore, a potential therapeutic target for the treatment of bone injury-related diseases.

A different study that analyzed the function of miR-9 in C2C12 mesenchymal cells further supported the role of miR-9 in osteoblast differentiation. By significantly decreasing the expression of DKK1 protein, but not of its mRNA, miR-9 stimulated alkaline phosphatase (ALP) activity and osteoblast mineralization, as well as the expression of several osteoblast marker genes, such as COL I (collagen I), OCN (osteocalcin), and BSP (bone sialoprotein) [107]. MiR-9 furthermore was detected as a tumor-secreted pro-angiogenic miRNA that promoted EC migration and tumor angiogenesis in vitro. Mechanistically, this was explained by interference with the expression levels of SOCS5, and thereby, the activation of the JAK-STAT pathway [108]. 

Moreover, altered expression of miR-9 was found during screening osteoarthritis cartilage involved in the control of tumor necrosis factor α (TNFα) expression. Here, miR-9 has been implicated as a key regulator in the process of endochondral ossification, since its expression varied significantly between the early and late stage of chondrocyte development [73,109]. Functional experiments in a mouse tibial plateau fracture model implicated that miR-9 and miR-181a significantly downregulated Bim concentration. Thereby, osteoclast survival was stimulated and the migration ability of osteoclasts was effected [110].

### 5.2. MiR-10a

The function of miR-10a during osteoblast differentiation and angiogenesis in vitro was analyzed in MC3T3-E1 and MUVEC (mouse umbilical vein endothelial cells) by the research group of Li et al., respectively [111]. Upon BMP2-induced osteoblast differentiation of MC3T3-E1 cells, miR-10a was downregulated. In contrast, when miR-10a was overexpressed, a suppressive effect on β-catenin and LEF1 expression could be demonstrated. Overexpression inhibited osteogenic differentiation, as demonstrated by reduced expression of the osteoblast-differentiation markers ALP, runt-related transcription factor 2 (RUNX2), Osterix (OSX), and distal-less homeobox 5 gene (DLX5). Moreover, it led to a decrease in MUVEC proliferation, migration and tube formation in combination with reduced concentrations of the angiogenesis-related genes VEGF, VE-cadherin, cyclin D1, and MMP2. As the canonical WNT/β-catenin signaling pathway was found to play an important role in osteogenic cell proliferation, differentiation, and bone regeneration, miR-10a offers a potential therapeutic target for the treatment of bone regeneration and bone-related diseases [112]. 

A further report referred to the role of miR-10a in regulating endothelial progenitor cell (EPC) senescence in the mouse [113]. Zhu et al. provided evidence that upon upregulation of miR-10A and miR-21, Hmga2 (High-mobility group AT-hook 2) expression is gradually decreased during aging in bone-marrow cells that were enriched for EPCs. Suppression of miR-10A* and miR-21 in aged EPCs, on the other hand, increased Hmga2 expression and improved EPC angiogenesis in vitro and in vivo. This could be demonstrated by rejuvenated EPCs, which resulted in decreased senescence-associated β–galactosidase expression, increased self-renewal potential and decreased p16Ink4a/p19Arf expression. In conclusion, the study demonstrates that the miR-10A*/miR-21–Hmga2–P16Ink4A/P19Arf axis controls EPC senescence and angiogenesis and may represent a potential therapeutic intervention target for improving EPC-mediated angiogenesis and vascular repair. 

### 5.3. MiR-10a/10b

Hassel et al. investigated the function of miR-10 in zebrafish blood vessel formation. During embryogenesis, the knockdown of miR-10a/10b impaired blood vessel outgrowth due to an altered tip cell differentiation behavior, and led to defects of intersegmental vessel growth by modulating fms-related tyrosine kinase 1 (flt1) levels post-transcriptionally [114]. However, as the knockdown of flt1 did not fully rescue the angiogenic phenotypes in miR-10 mutant zebrafish, as well as in miR-10-deficient HUVECs, the authors concluded that in ECs, flt1 could not represent a direct exclusive target of miR-10. They provided evidence that miR-10a/10b regulated angiogenesis in a Notch-dependent manner by directly targeting mib1 (mindbomb E3 ubiquitin protein ligase 1) in zebrafish ECs. Inhibition of mib1 and Notch signaling partially rescued the angiogenic defects in miR-10 morphants, suggesting that the observed angiogenic defects in miR-10a/10b morphants are caused by up-regulation of Notch signaling [115]. 

### 5.4. MiR-20a

As a member of the extensively studied miR-17-92 cluster with prominent roles in tissue and organ development, the role of miR-20a was elucidated during osteogenesis by Zhang et al. [116]. They disclosed that, together with several osteoblast markers (BMP2, BMP4, RUNX2, OSX, OCN and OPN), miR-20a was upregulated during osteogenic differentiation of hBM-MSCs derived from bone marrow from differently aged persons. Adipocyte markers, however, such as PPARγ and the osteoblast antagonists, BAMBI and CRIM1, were down-modulated. By introducing miR-20a mimics and lentiviral-miR20a-expression vectors into hBM-MSCs, they verified that miR-20a enhances osteogenic differentiation. Simultaneous direct interaction with all the aforementioned positive and negative effectors of BMP/RUNX2 signaling could be confirmed.

In a report by Doebele et al., miR-20a was investigated with respect to its cell-intrinsic angiogenic activity in EC, as different members of the miR-17-92 cluster that are highly expressed in tumor cells were found to be expressed at increased levels during ischemic conditions [117]. They determined that in vitro overexpression of miR-20a (together with miR-17, -18a, -19a) rigorously inhibited EC sprout formation, whereas their inhibition using antagomiR treatment led to an increase of spheroid sprouting (irrespective of miR-19a). Interestingly, in vivo matrigel plug assays employing antagomiRs for miRs-17-20a as inhibitors demonstrated enhanced angiogenic sprouting, but in contrast to in vitro results, they were unaltered in tumor angiogenesis, indicating context-sensitive regulation. In particular, the pro-angiogenic target gene Janus kinase 1, but also the cell cycle inhibitor p21 and the S1P receptor EDG, were shown to be downregulated by miR-17/20a. 

Deng et al. provide evidence that miR-20a and miR-31 serve as stimulators of angiogenesis [118]. As an underlying mechanism, they found that the expression of both miRNA molecules is upregulated via AKT and ERK signals that are themselves activated by the angiogenic factor VEGF. As target genes, miR-20a and miR-31 were found to directly associate with the 3´-UTR of the tumor necrosis factor superfamily-15 (TNFSF15) gene, thereby clarifying its by then unknown mechanistic role in vascular homeostasis. TNFSF15 is expressed in ECs of mature vasculature, and is a known inhibitor of angiogenesis. Interestingly, VEGF-stimulated downregulation of TNFSF15 could be attenuated by treatment of HUVECs with AKT inhibitor LY294002, leading to reduced miR-20a and miR-31 levels, while ERK inhibitor U0126 prevented VEGF-induced expression of miR-20a only. In contrast, inactivation of either ERK or AKT signals restored TNFSF15 gene expression and elevated miR-20a or miR-31 levels which led to enhanced capillary-like tube formation in an in vitro angiogenesis assay.

### 5.5. MiR-26a/b

Luzi et al. investigated and confirmed an important function of miR-26a during human adipose-derived MSCs (hADSCs) differentiation towards the osteogenic lineage induced by treatment with dexamethasone, ascorbic acid, and beta-glycerol phosphate [119]. Upon inhibition of miR-26a by antisense RNA, upregulation of the transcription factor SMAD1—which was predicted in silico—and its regulated osteogenic differentiation marker genes could be observed in treated osteoblasts. In a follow-up study of Luzi et al., these results were extended to include the interaction between menin and miR-26a as regulators of osteogenic differentiation in hADSCs [120]. Menin is a presumable transcriptional regulator that modulates mesenchymal cell commitment to the myogenic or osteogenic lineages. It is encoded by the *MEN1* oncosuppressor gene which causes the multiple endocrine neoplasia type-1 syndrome. The results demonstrated orchestrated down-regulation of *MEN1* mRNA and miR-26a, with a consequent up-regulation of SMAD1 protein in hADSCs. 

Su et al., however, reported an opposing role of miR-26 in hBM-MSC during osteogenic differentiation, suggesting distinct post-transcriptional regulation of tissue-specific hMSC differentiation [121]. Using bioinformatics and functional assays, they confirmed that miR-26a directly regulates *SMAD1*, but added GSK3β as a target to regulate BMP and WNT signaling pathways. The distinct activation pattern and comparative analysis revealed that miR-26a significantly inhibited *SMAD1* to suppress BMP signaling for interfering with the osteogenic differentiation of hADSCs, whereas it targeted on GSK3β to activate WNT signaling for promoting osteogenic differentiation of hBM-SCs. Overall, they concluded that the BMP pathway was more essential for promoting osteogenic differentiation of hADSCs, whereas WNT signaling was enhanced more potently and played a more important role than BMP signaling in osteogenic differentiation of hBM-SCs. In conclusion, although miR-26a enhances osteogenic differentiation in both cell types, different signaling pathways were employed in hBM-MSCs and hADSCs. 

In to addition unrestricted somatic stem cells (USSC), the studies of Trompeter et al. investigated a rare population in human cord blood with respect to osteogenic differentiation. Gene expression profiling of two different USSC cell lines (SA5/73 and SA8/25) identified, among other candidates, miRs-26a/b and miR-29b to be consistently upregulated during osteogenic differentiation [122]. As osteo-inhibitory targets of these miRNAs, CDK6 and HDAC4 were evaluated that were downregulated during osteogenic differentiation of USSC, whereas SMAD1 was found as an osteo-promoting target. During osteogenic differentiation of USSC or following ectopic expression of miR-26a/b, SMAD1 exhibited an unchanged expression level, however. 

Entangling of miR-26a in pathological and physiological angiogenesis was investigated by Icli et al. in ECs [123]. They studied the effects of modifying the expression of BMP/SMAD1 signaling. Upregulation of miR-26a led to EC cycle arrest, inhibited EC migration, sprouting angiogenesis, and network tube formation in matrigel. Upon inhibiting miR-26a expression, a contrasting phenotype could be detected. At the molecular level, Icli et al. demonstrated direct binding of miR-26 to the 3′-UTR of *SMAD1* thereby reducing its mRNA levels, which subsequently suppressed *ID1* expression and increased *p21WAF/CIP* and *p27* protein expression. 

### 5.6. MiR-29b

MiRNA profiling of MC3T3 preosteoblastic cells derived from fetal mouse calvaria and differentiated to osteoblasts led to the identification of miR-29b, among other members of the miR-29, miR-let-7, and miR-26 families by Li et al. [124]. Versatile effects of miR-29b were found to promote osteoblastogenesis at multiple stages as a key regulator. One mechanism pursues the silencing of negative regulators of osteogenic differentiation, such as TGF-β3, HDAC4, ACTVR2A, CTNNBIP1, and DUSP2 that involve particularly the osteogenic function of RUNX2, as well as the SMAD, ERK, p38 MAPK, and WNT signaling pathways. A second path seeks the suppression of extracellular matrix protein synthesis relevant to bone development (such as COL1A1, COL5A3, and COL4A2) to preserve the differentiated phenotype during mineralization of mature osteoblasts. This alternative mechanism seems to enhance mineral deposition and to prevent fibrosis.

Rossi et al. also disclosed a link of miR-29b to osteoclastogenesis and proposed it for the treatment of multiple myeloma-related bone disease as its expression declined increasingly during human osteoclast differentiation and affected proper bone resorption [125]. Several findings indicated that miR-29b is a negative regulator of human OCL differentiation and activity. Thus, lentiviral transduction of miR-29b into OCLs was associated with diminished tartrate acid phosphatase expression, lacunae generation, and collagen degradation. Attenuated resorptive osteoclast capabilities, due to miR-29b inhibition of proteolytic enzymes, were documented by reduced cathepsin K, MMP-9, and MMP-2 expression. Overall, downstream phenotypic effects along the M-CSF and RANK-L axes that led to impaired action of the master transcription factor NAFTc-1 were explained by miR-29b targeting of c-FOS.

Zhang et al. found inhibitory activity of miR-29b on VEGF secretion via the anti-angiogenic cytokine TNFSF15 (VEGI; TL1A) in the mouse EC line bEnd.3, which defines a new angiogenesis-related signaling pathway [126]. In contrast, down-modulation of TNFSF15 activity by a specific siRNA against its receptor DR3/TNFRSF25, or a neutralizing antibody against TNFSF15, reinstated VEGF generation but suppressed miR-29b expression. TNFSF15-enhanced activation of the JNK-GATA3 signaling pathway was furthermore able to stimulate miR-29b expression but silenced VEGF production, as demonstrated by a specific JNK inhibitor or siRNA.

Li et al. described an anti-angiogenic and anti-tumorigenic role for miR-29b by the regulation of AKT3 expression [127]. AKT is known to induce tumor vascularization via VEGF, and cancer cell activity via c-MYC arrest, in breast tumor. In vitro and in vivo ectopic expression of miR-29b therefore blocked angiogenesis, as well as tumor cell formation, evidencing it as a potential useful anti-cancer therapeutic agent.

### 5.7. MiR-31

Granchi et al. detected miR-31 by profiling miRNA expression during osteogenic differentiation and mineralization of hBM-MSCs that were derived from three individual donors [128]. As an identified direct target gene, miR-31 differentially modulated the expression of the bone-specific transcription factor OSX during osteogenic differentiation [129]. This could be demonstrated by an inverse miRNA-target expression ratio in osteosarcoma cell lines and an increase in OSX expression upon specific miR-31 inhibition.

Deng et al. provided evidence that the expression of miR-31 increasingly declined during the osteogenic differentiation of hBM-MSC cells [130]. This regulation coincided with increased ALP activity, mineralization of hBM-MSC cultures and expression of the osteogenic transcription factors OPN, BSP, OSX, and OCN, with the exception of RUNX2. Mechanistically, they uncovered a RUNX2, SATB2, and miR-31 regulatory feedback loop that determined hBM-MSC differentiation using inhibitors and mimics of miR-31. RUNX2 directly regulates miR-31 expression levels which itself controls the translation of SATB2 protein.

In a study of Suarez et al. that investigated TNF-mediated induction of endothelial adhesion molecules, the expression of miR-155, -31, -17, and -191 were found to be increased without a change of miR-20a, -222, and -126 levels in HUVECs [84]. By miRNA target prediction algorithms, the 3´-UTR of E-selectin was identified as target molecule, which was verified experimentally by reporter assays using miR-31 mimics. miR-31-mediated regulation of E-selectin expression not only regulates binding of neutrophil granulocytes to HUVECs, but is also involved in inhibition of angiostatin-induced angiogenesis by affecting cell migration [131].

An angiogenesis-related function of miR-31 reported by Deng et al. has already been described above in the subsection of miR-20a [118].

### 5.8. MiR-34a

Opposing roles for miR-34a in differentiation of hMSC towards osteoblast have been reported. Chen et al. identified miR-34a in a microarray screening, and stated that it inhibits osteoblast differentiation in hMSC and in vivo bone formation in a preclinical model of heterotopic bone formation in mice [132]. Thus, when miR-34a was overexpressed in hMSC, it inhibited early and late OB commitment, as well as differentiation and hMSC proliferation, while anti-miR oligonucleotide treatment reversed these effects. In addition to several cell cycle regulator and cell proliferation proteins (including CDK4, CDK6, and Cyclin D1), JAGGED1, a NOTCH1 receptor ligand, was elicited as a target gene of miR-34a that was regulated at both the transcriptional and translational levels, as determined by RNA interference. JAGGED1 has previously been implicated in human bone biology, as its deficiency causes skeletal abnormalities in the Alagille syndrome.

Kang et al., however, claimed that miR-34a-5p-induced activation of the Notch signaling pathway is a positive regulator of glucocorticoid-mediated osteogenic differentiation of hMSCs [133]. They demonstrated dexamethasone-inhibited osteoblastic differentiation of murine BM-MSC via miR-34a-5p-mediated gene silencing of coincidently identical target genes, as published by Chen et al. Differences in both reports were discussed to be due to different roles of the Notch signaling pathway in osteogenic differentiation of hMSCs that were derived from different species.

A more recent publication by Fan et al. found miR-34a upregulation during osteogenic differentiation of hADSCs [134]. Elevated levels of miR-34a in hADSCs promoted mineralization, ALP activity, and the expression of the key regulatory osteogenic transcription factor *RUNX2* by targeting the retinoblastoma binding protein 2 (RBP2); furthermore, heterotopic bone formation was enhanced in vivo. Expression of *NOTCH1* and *Cyclin D1* genes, that were also involved in this coregulatory network, were found to be downregulated on the other hand, which facilitates cell cycle exit [135,136]; this is the consequence of suppressed proliferation but enforced terminal maturation of osteoblasts by RUNX2.

When investigating the role of miR-34a in glucocorticoid-induced osteonecrosis of the femoral head (GIOFH), Zha et al. verified the findings of Kang et al., but extended this knowledge [137]. By investigating dexamethasone-treated rat subjected to miR-34a-overexpressing lentiviruses, decreased blood vessel development was observed, indicating that VEGF presents a regulatory target of miR-34a. In vitro, miR-34a overexpression enhanced the inhibitory effects of dexamethasone on the viability and activity of ECs and downregulated VEGF protein expression levels.

A study by Zhao et al. investigated the angiogenic role of miR-34a in EPCs derived from adult male Spraque-Dawley rats [138]. The rationale behind this project was the previous implication of miR-34a in targeting silent information regulator 1 (*SIRT1*), provoking cell cycle arrest or apoptosis. The results confirmed the inhibitory effects of raised miR-34a expression levels on *SIRT1* and, thus, on EPC-mediated angiogenesis by inducing senescence. Mechanistic causes could be found in increased acetylated levels of the FOXO1 transcription factor, regulated by *SIRT1,* which could be demonstrated by knockdown of *SIRT1*. The angiogenesis-promoting role of miR-34a and Sirt1 in this context seems to lie in its previously discovered function in vascular endothelial homeostasis by preventing stress-induced senescence in health and disease [139].

A negative regulatory role for miR-34a could also be documented by Kumar et al. in tumor angiogenesis in head and neck squamous cell carcinoma (HNSCC) [140]. MiR-34a expression was markedly decreased in HNSCC tumors and cell lines, and ectopic expression of the miR-34a therefore affected cell proliferation and migration in HNSCC cell lines and in a SCID mouse xenograft model. These effects seemed to be mediated by the regulation of survivin expression via the transcription factor E2F3a that is critical for cell cycle progression. Furthermore, tumor angiogenesis was found to be dysregulated by interference of miR-34a with VEGF secretion in tumor cells, as well as EC proliferation, migration, and tube formation, by downregulating a number of key proteins including E2F3, SIRT1, survivin, and CDK4.

### 5.9. MiR-92a

The autosomal dominant Feingold syndrome that is characterized by microcephaly, short stature, and digital anomalies was identified in individuals carrying hemizygous deletions of the miR-17-92 cluster [172]. To dissect this complex phenotype that could be partially mirrored in mice deficient of the miR-17-92 cluster, and due to postnatal lethality, a single miR-92a targeted mouse line was established by Penzkofer et al. [173]. Surviving mice exhibited reduced body weight and skeletal defects represented by reductions in body length, skull, and tibia length, as well as metacarpal bone size; these defects were presumably also the result of osteoblast proliferation and differentiation phenotypes, as found in hemizygous miR-17-92 mouse mutants [174]. In contrast, however, single miR-92a deletion does not cause low bone mineral density attributed to reduced type-I collagen mRNA, ALP activity, and mineralization ability. Phenotypic differences could be explained by the design of the genomic deletion of miR-92a that partially attenuated expression levels of miR-18a in skeletal tissue. The direct molecular targets of miR-92a responsible for regulation of osteogenesis have not yet been elicited.

A recent study of Mao et al., however, identified aggrecanase-1 and aggrecanase-2 (ADAMTS4/5) as direct miR-92a targets in chondrogenic hMSCs and human chondrocytes by reporter assays [175]. These two members of the ADAMTS family represent MMPs that are important for normal chondrocyte differentiation, but which also promote the progression of osteoarthritis by cartilage degeneration. In comparison to normal cartilage, real-time-PCR analyses also revealed higher miR-92a-3p expression levels in chondrogenic hMSCs, whereas markedly reduced miRNA expression could be detected in ostearthritis cartilage. Moreover, miR-92a-3p-modified expression of ADAMTS-4/5 could be downregulated by IL-1β transfected primary human chondrocytes. The study was preceded by previous findings about miR-92a-3p upregulation in hADSC-derived chondrocytes and chondrogenic hMSCs, as well as osteoarthritis cartilage, where histone deacetylase 2 (HDAC2) was identified as a target gene of miR-92a-3p [176,177]. 

MiR-92a was first reported as a part of the miR-17-92 cluster by Bonauer et al., and was found to be abundant in human ECs [178]. MiR-92 was established as a suppressor of angiogenesis which targets the expression of several proangiogenic proteins, including the integrin subunit alpha 5 (ITGA5) that is well known for severe vascular defects in gene targeted mice. Systemic administration of miR-92a antagomiR therefore improved the growth of blood vessels and functional recovery of damaged tissue in mouse models of limb ischemia and myocardial infarction, possibly by indirectly inhibiting apoptosis [58]. Thus, miR-92a may serve as a valuable therapeutic target in the setting of ischemic disease. 

Daniel et al. further elaborated the function of miR-92a upon re-endothelialization and neointimal formation after wire-induced injury of the femoral artery in mice [144]. By using specific LNA (locked nucleic acids)-based antimiRs as well as miR-92a-deficient ECs in the mouse, they found enhanced re-endothelialization and inhibited neointimal formation induced by de-repression of the miR-92a targets integrin a5 and sirt1 [179]. Thus, an important role can be attributed to miR-92a in blood vessel regeneration in ischemic tissues and after vascular injury. 

In a subsequent study, Zhang et al. investigated the ability of miR-92a to influence apoptosis and angiogenesis in ECs in the presence of oxidative stress [180]. It was previously determined that senescent ECs that undergo apoptosis, and which are frequent in ageing and atherosclerosis, have diminished miR-92a levels [181]. The results provided evidence that pre-miR-92a treatment of HUVECs prevented oxidative stress-induced apoptosis in EC, whereas capillary tube formation i.e., angiogenesis, was maintained. Mechanistically, it was determined that pre-miR-92 directly repressed PTEN, and thereby, activated the AKT signaling pathway that regulates EC apoptosis and angiogenesis. 

In a study of Kalinina et al., paracrine effects of miR-92a on hMSCs were studied [143]. Conditioned medium of hMSCs transfected with pre-miR-92a prevented tube formation by HUVECs, which could be attributed to significantly lower secretion of hepatocyte growth factor (HGF) and angiopoetin-1 independent of VEGF secretion. HGF is a factor required for stimulation of proliferation of ECs, whereas angiopoietin-1 regulates vessel stabilization and maturation. As neither gene was predicted as direct miR-92a targets, these still have to be identified. Restoration of tube formation was achieved by replenishment of HGF, but not with anti-miR-92a treatment of hMSCs. This led to the conclusion that miR-92a suppresses hMSC-induced angiogenesis by downregulating the secretion of HGF and, therefore, is involved in the control of anti-angiogenic activities in hMSCs.

### 5.10. MiR-125b

MiR-125b is an early discovered miRNA that plays a key role in cellular functions. Experiments by Mizuno et al. using BMP-4-induced or exogenous miRNA-transfected murine MSC ST2 cells found miR-125b to inhibit osteoblastic (and adipogenic) differentiation through modulating cell proliferation [146]. Further experiments with transfection of siRNA identified ErbB2 as a target gene. miR125b-gene targeted mice, generated by Lee et al., identified further supported the important function of miR-125b in regulating stem cell directional differentiation, but did not reveal its molecular role [182]. 

Decreasing levels of miR-125b were also identified by Huang et al. during the differentiation of C3H10T1/2 cells, and reporter gene assays led to the identification of a putative target binding site in the 3´-UTR of the Cbfβ gene, a master regulatory gene of osteogenesis [148]. Therefore, silencing of miR-125b increased the mRNA levels of Cbfβ and of osteoblastic marker genes ALP, OCN, and OPN. Conclusively, RUNX2 is considered as an indirect target of miR-125b as well.

Transient expression of miR-125b expression in hypoxic VEGF-or or bFGF stimulated ECs was moreover shown by Muramatsu et al. to directly bind and block the translation of vascular endothelial (VE)-cadherin mRNA and therefore inhibit in vitro tube formation by ECs [150]. Thus, miR-125b may be tested in tumor therapy for inducing disruption of blood vessel formation.

### 5.11. MiR-135b

During the osteogenic differentiation of several USSC, miR-135b was identified as the most consistently down-regulated candidate by Schaap-Oziemlak et al. [151]. Retroviral overexpression resulting in decreased mineralization confirmed a function of miR-135b in osteogenesis of USSC. Furthermore, quantitative RT-PCR analysis of USSC that overexpressed miR-135b showed decreased expression of the bone mineralization markers IBSP and OSX. 

In a profiling study by Xu et al. that investigated the exosomal content of hBM-MSCs during osteogenic differentiation, miR-135b was found to be significantly increased [183]. Bioinformatic analysis led to the conclusion that several important pathways related to osteoblastic differentiation were engaged, and that exosomal miRNA is a regulator thereof. 

By establishing hypoxia-resistant multiple myeloma (MM) cells through exposure to chronic hypoxia, Umezu et al. presumably identified a new mechanism of hypoxia-induced angiogenesis targeting the FIH-1/HIF-1 signaling pathway via exosome-contained miRNAs [152,184]. In contrast to the transiently hypoxia-upregulated miR-210 transcriptional levels which decline gradually under normoxic conditions, miR-135b levels were maintained high, even under normoxic conditions. By delivering exosomes to HUVECs, miR-135b is enabled to target the factor-inhibiting hypoxia-inducible factor-1 gene (FIH-1) that encodes an asparaginyl hydroxylase enzyme which inhibits HIF-1a. The positive correlation between miR-135b, HIF-1a, and microvessel density was initially identified by Zhang et al. in a HNSCC model [185]. As a result, endothelial tube formation could be promoted under hypoxic as well as normoxic environments.

### 5.12. MiR-181a

Among other functions, the miR-181 family has been implicated in genetic regulation of early hematopoiesis and lymphangiogenesis [60,186,187]. Microarray analysis of human HCS-2/8 cells led to the characterization of miR-181a. Sumiyoshi et al. accredited miR-181a with an important function in the maintenance of cartilaginous metabolism by a negative feedback system employing repression of the CCN family member 1 (CCN1) and aggrecan (ACAN) genes, which are both known to be involved in chondrocyte differentiation [154].

In another study, the examination of synovial fluid cells in a mouse model of tibial plateau fractures led to the detection of two downregulated miRNAs, miR-9 and miR-181a [110]. Cbl, an important E3 ubiquitin ligase for bone resorption that was tested as a putative target gene of miR-9 and miR-181a, elicited increased amounts of ubiquitinated Bim, a pro-apoptotic gene in mouse primary osteoclast cells. Therefore, Wang et al. concluded that upregulated Cbl might regulate the survival rate of primary mouse osteoclast cells, as previously Cbl-dependent apoptosis via ubiquitinated Bim was reported [188].

Sun et al. implicated miR-181a as a potential oncomiR (cancer-associated miRNA) in the angiogenesis and metastasis of chondrosarcoma in a xenograft mouse model [156]. They found that miR-181 increases VEGF and MMP1 expression, as well as CXCR4 signaling, via negatively regulating RGS16 (regulator of G-protein signaling 16) under hypoxic cell culture conditions. RGS16 is an inhibitor of CXCR4 which regulates, upon amplified signaling, angiogenesis, invasion, and metastasis in chondrosarcoma. The therapeutic usefulness of this mechanism was proven by miR-181a antagomiR treatment, which decreased proangiogenic gene expression as well as tumor growth and metastasis in a xenograft mouse model.

### 5.13. MiR-195

Together with miR-497, miR-195 was downregulated in a microarray screen of primary hMSCs performed by Almeida et al. [158]. hMSCs underwent induced osteogenic differentiation with the aim of identifying candidates that are capable of contributing to bone fracture repair. Osteogenic markers were therefore found to be diminished upon overexpression or increased upon inhibition of miR-195 in hMSCs. Using the chicken CAM assay, studying the paracrine effects of hMSCs, the authors furthermore demonstrated decreased blood vessel formation in vivo. VEGF was identified as the target gene that mediates this phenotype, at least in part. MiR-195 interacted with the VEGF 3´-UTR in bone cancer cells and also regulated mRNA and protein expression levels.

Reduced expression of miR-195 furthermore resulted in increased angiogenesis and metastasis in HCC tissues, whereas either loss-of-function or gain-of-function abrogated the ability of HCC cells to migrate and induce capillary tube formation of ECs, as reported by Wang et al. [159]. In xenograft tumors, upregulated miR-195 expression provoked reduced microvessel densities and the formation of metastases. Detailed molecular investigations disclosed VEGF and the prometastatic factors VAV2 and CDC42 as direct targets of miR-195, which could be proven by mirroring the phenotype either by knockdown or overexpression of these target genes. The group also demonstrated that higher VEGF levels due to miR-195 down-regulation promoted EC-mediated tumor angiogenesis by the involvement of VEGF receptor 2 signaling.

### 5.14. MiR-200b

miR-200b was identified by microarray screening as a miRNA that exhibits downregulated expression upon exposure of osteoblasts to collagen and to silicate-based periodontal grafting material (PerioGlas, P-15) that is used to promote bone formation [189]**.** Rønbjerg et al. demonstrated miR-200b as a potent regulator of the target gene ZFHX1B via direct interaction [190]. ZFHX1B is a transcriptional repressor involved in the regulation of the TGFβ signaling pathway and epithelial-mesenchymal transitions by E-cadherin, a mediator of cell-cell adhesion, in mesenchymal cells. 

In addition to the known communication of interacting cells by the exosomal release of miRNAs (e.g., mir-135b [153,191]), Fan et al. discovered angiogenic-osteogenic cell coupling and reciprocal interactions via gap junctions [161,192]. They provided evidence that in a direct co-culture, miR-200b was transferred in a TGF-β-stimulated process from rat BM-MSCs to HUVECs through gap junctions formed of connexin 43. By this transfer and decrease of miR-200b, VEGF-A-induced expression enhanced osteogenic differentiation in BM-MSCs. In HUVECs, increasing miR-200b levels down-modulated ZEB2, ETS1, KDR and GATA2 transcription factors, which led to a decline of the angiogenic potential of HUVECs, in contrast. In vitro angiogenesis in this co-culture could therefore be partially rescued by employing the TGF-β inhibitor SB431542 or TGF-β-neutralizing antibody. These findings could provide a new strategy for cell-based bone regeneration. 

In lung epithelial carcinoma cells (A549 cells), direct negative regulation of VEGF, VEGFR1 (Flt-1) and VEGFR2 (KDR) by binding of miR-200b to the corresponding 3´-UTR could be demonstrated by Liu et al. [193]. This interaction could be furthermore confirmed in an in vitro angiogenesis assay by transfection of HUVECs, which resulted in reduced capillary tube formation and significantly reduced VEGF-induced phosphorylation of ERK1/2. In addition, miR-200b targets the Ets-1 transcription factor, that might concomitantly downregulate VEGFR2, as found in human mammalian epithelial cells by Chan et al. [194]. Thus, miR-200b may be used as a therapeutic angiogenesis inhibitor.

### 5.15. MiR-210

Upregulated expression of miR-210 was detected in BMP-4-induced osteoblastic differentiation of murine stromal BM-MSC ST2 cells by Mizuno et al. [163]. Transfection experiments of sense and antisense miR-210 therefore promoted or repressed osteogenesis, respectively. As a target gene mediating the positive regulation, the activin A receptor type-1B (AcvR1b) gene was elicited that effects the TGF-β/activin signaling pathway negatively.

Studies of Fasanaro et al. proved that the expression of miR-210 progressively increases upon exposure to hypoxia. The overexpression of miR-210 in HUVECs using miRNA mimics stimulated the formation of capillary-like structures and enhanced *VEGF*-induced cell chemotaxis [165,195,196]. Thus, miR-210 up-regulation is a crucial hypoxia response element of ECs, affecting cell survival, migration, and differentiation and might participate in the modulation of the angiogenic response to ischemia [99,197,198]. As a consistently reported target gene mediating these effects, Ephrin-A3 receptor tyrosine kinase ligand (*EFNA3*) was validated. *EFNA3* was previously shown to play a role in the regulation of angiogenesis and VEGF signaling during vascular development and remodeling via EFNA1/EphA2 interaction [199,200]. Overexpression of an Ephrin-A3 allele that is not targeted by miR-210 therefore prevented miR-210-mediated stimulation of tube formation and EC migration. 

In its function of promoting osteoblast differentiation by increased VEGF, ALP and OSX expression in rat MSCs and suppression of adipocyte differentiation, due to decreased PPAR-γ in vitro, miR-210 was also implicated in the regulation of postmenopausal osteoporosis [164]. Although the exact mechanism still needs to be elaborated, Liu et al. also found that HIF-1α and VEGF expression was increased in 17β-estradiol (E2)-treated osteoblasts.

### 5.16. MiR-222

Yan et al. identified miR-222-3p as a negative regulator involved in osteogenic differentiation of hBM-MSCs [166]. Enhancement of miR-222-3p function in hBM-MSCs was blocking protein levels of SMAD5 and RUNX2. miR-222-3p-specific inhibition via lentivirus infection, in contrast, led to their enhanced expression and increased phosphorylation of Smad proteins (1, 5, 8) that are responsible for expression of osteogenic genes. Thus, in addition to the Smad5-RUNX2 signaling pathway elevated levels of the osteoblast markers OSX, ALP, and OC, as well as increased matrix mineralization, could be detected.

The role of miR-222-3p in c-Src-mediated regulation of osteoclastogenesis was proven by Takigawa et al. [167]. Depending on the use of miR-222-3p inhibitor or mimics in RAW264.7 pre-osteoclastic cells, either upregulation, or downregulation of the mRNA expression levels of osteoclast marker genes NFATc1 or TRAP, were observed, respectively. Inhibition of of c-Src activity and activation of osteoclastogenesis via miR-222-3p was implemented by increased amounts of RANKL-induced expression of TRAP and cathepsin K protein levels. Thereby, the number of multi-nucleated osteoclasts and their pit formation was reduced.

Poliseno et al. investigated the role of miR-221 and miR-222 during in vitro angiogenesis [168]. Both miRNAs were identified among 15 upregulated miRNAs that allegedly target receptors of angiogenic factors in a large-scale screen of HUVECs. Mechanistically, they were found to post-translationally modify the angiogenic effects of stem cell factor (SCF) by targeting its receptor, c-KIT. Together with other angiogenic growth factors, such as VEGF, bFGF, or HGF, SCF is able to stimulate proliferation and migration of ECs and induce capillary-like tube formation when performing in vitro angiogenesis assays [201]. Consistently, tube formation, wound healing, or cell migration was suppressed in miR-221/miR-222– transfected and SCF-treated HUVECs.

### 5.17. MiR-424

miR-424 was identified by differential screening in a miRNA microarray of isolated primary hMSCs from four individuals that were, or were not, osteo-differentiated using osteogenic differentiation medium. In combination with miR-31, miR-106a, and miR-148a, Gao et al. found that miR-424 was suppressed, and predicted it to target RUNX2, CBFB (core-binding factor, beta subunit), and BMPs [169]. In a similar experimental setting of Vimalraj et al., miR-424, together with miR-106a, miR-148a, let-7i and miR-99a, were detected as hMSC-specific miRNAs that were found to be expressed only in undifferentiated hMSCs [170]. Here, bioinformatics analysis mostly predicted the MAPK, WNT, and insulin signaling pathways as targets.

A recent study of Li et al. elucidated further specific functional mechanisms of miR-424 during bone formation and oxidative stress [171]. It was reported that miR-424 was downregulated by the transcription factor FOXO1 using consensus binding sites in the promoter. Subsequently, via miR-424 mediated upregulation of FGF2 under oxidative stress, proliferation and osteogenic differentiation of hMSCs was accomplished. Uncovering the miR-424/FGF2 pathway revealed a new mechanism how FOXO1 promotes bone formation and could potentially enhance bone repair. 

miR-424 was furthermore recognized by Gosh et al. as an important hypoxamiR in human ECs by revealing a novel pathway for HIF regulation [101]. Under hypoxic conditions levels miR-424 or its rodent homolog, mu-miR-322, were found to be elevated in ECs and in ischemic tissues undergoing vascular remodeling in an experimental myocardial infarction rat model. It was elicited that the target of miR-424 is represented by cullin 2 (CUL2), which serves the purpose of stabilizing the hypoxic transcription factor HIF-1α. CUL2 is an essential component for assembling the ubiquitin ligase system, normally leading to its continuous degradation. MiR-424 expression was experimentally evaluated to be regulated by the transcription factor PU.1 that itself was found to be regulated by RUNX-1 and C/EBPα transcription factors. Ectopic expression of miR-424 in retrovirally-transduced HUVECs therefore stimulated angiogenesis in vivo in athymic mice that were subcutaneously implanted.

## 6. Outlook and Future Directions: MiRNAs in Therapeutic Applications

Bone is a highly vascularized tissue, and is thus reliant on the coordinated interaction between osteogenesis and angiogenesis which occurs between osteoblastic and ECs during development, remodeling and regeneration of the skeletal system [22,27,202]. Deciphering the molecular nature of the mechanisms that couple bone formation to blood vessel formation should therefore be of great interest to enhance bone formation capabilities in vitro and in vivo [203]. These specific underlying mechanisms are gaining growing attention under physiologic and pathologic conditions such as fracture healing, prevention of osteoporosis, tissue engineering or bone regeneration. Thus far, mostly a combination of different growth factors controlling osteogenesis and angiogenesis (e.g., such VEGF, angiopoietins, BMPS, RUNX2) have succeeded in achieving bone formation to a certain degree [33,204,205,206]. Nevertheless, each of these factors has individual roles at certain stages of development, and may disturb the subtle orchestration of required regulation steps.

MicroRNAs provide potent modifiers which coordinate a broad spectrum of biological processes. In contrast to transcription factors, single miRNAs may only modestly affect individual mRNA target expression. However, on the one hand a specific target mRNA can experience increased repression if it has multiple binding sites in the 3′-UTR. On the other hand, the same 3′-UTR of a gene can be targeted by many different miRNAs simultaneously that intervene with its regulation of expression [207]. Bioinformatic analysis of target prediction databases, such as TargetScan, miRanda and Pictar, suggest that a single average miRNA is capable of modulating up to several hundred target genes by perfect or imperfect base-pairing in combination with tissue-specific expression [208,209,210,211]. Additionally, by feedforward or feedback loops that form a regulatory network, miRNA effects can be amplified [212]. And it is estimated that approximately one-third of genome-encoded proteins are effected by miRNA regulation [213]. Therefore, these regulatory RNAs which control multiple endogenous signaling processes simultaneously possess a unique capacity to interfere with all cellular processes and have become an important tool in biological and medical research. As the application of miRNA-based methods for the treatment and monitoring of different pathological conditions is constantly becoming more prevalent, their therapeutic engagement could establish a refined method to stimulate inartificial bone development. 

There is increasing evidence that miRNAs play important roles in controlling osteogenesis and angiogenesis. One study identified mutations in miR-2861 in two related adolescents that likely contributed to primary osteoporosis [214]. miR-2861 regulates osteoblast differentiation by targeting HDAC5, which enhances RUNX2 degradation. Moreover, a recent study showed accelerated osteoblast differentiation of hMSCs in a three-dimensional scaffold in vitro through manipulation of miR-148b and miR-489 expression [215]. Several studies also explored the use of miRNA mimicking or inhibitory agents for bone regeneration in animal studies. However, only a few studies have found that miRNAs are positive regulators of these processes. As outlined, miR-29b has been reported to promote osteoblast differentiation by targeting several well characterized inhibitors of bone formation in vitro [124]. Single site-specific delivery of miR-29b into a two-week post fracture callus by Lee et al. significantly improved mouse femoral fracture healing [216]. This was documented radiographically by a decrease of callus width and area; histomorphometrical and micro-computed tomographical analyses demonstrated increased bone volume fraction and bone mineral density of the callus. A single report further delineated the use of miRNAs as a therapeutic strategy to modulate angiogenesis-osteogenesis coupling during bone regeneration or repair in a subcutaneous assay in the mouse. The studies of Li et al. preferred miR-26a over miR-21 and miR-29b, which were all identified as the most potent candidates to mediate both angiogenesis and osteogenesis by microarray profiling of primary osteoblasts [217]. miR-26a was found to be a factor that is upregulated in newly-formed bone, and itself stimulates the expression of osteoblast-specific makers RUNX2, ALP, and OCN, mineralization of osteoblasts as well as VEGF secretion in murine primary BM-MSCs and in MC3T3-E1 cells in vitro. The enhancement of miR-26a expression in vivo by transfection of BM-MSCs with miR-26a mimicking agents led to complete repair of a critical-size calvarial bone defect, mainly due to simultaneously regulating endogenous angiogenesis-osteogenesis coupling. To date, its ability to integrate multiple signaling pathways thus makes miR-26a an ideal candidate for regenerating bone by miRNA-based therapy. Murata et al. detected significantly decreased levels of miR-92a in patients with trochanteric fractures, and investigated its significance in a mouse femoral fracture model [142]. Systemic as well as local administration of antimir-92a via LNA-stabilized oligonucleotide increased callus volume and enhanced fracture healing in the early phase by promoting neovascularization in the mouse femur. Nevertheless, as outlined previously, the direct osteogenesis-related molecular targets for this mechanism, which offers therapeutic potential for repairing bone, still remain to be clarified. In addition to the basic osteogenic functions of miR-31, Deng et al. investigated the therapeutic potential of hAD-MSC combined with beta-calcium triphosphate scaffolds in repairing a rat critical-sized calvarial defect [130,217]. The group reported that a knockdown of miR-31 significantly enhanced the repair of the defect, as could be noticed by an increased bone volume and mineral density in combination with a decreased scaffold. As the molecular basis of the osteogenic differentiation and bone regeneration program, in vitro results using lentiviral expression constructs revealed a BMP-2-inducible regulatory loop between Runx2, miR-31, as well as the miR-31 target gene, AT-rich sequence-binding protein 2 (Satb2). As a negative modulator of angiogenesis, Yoshizuka et al. locally administered miR-222 inhibitor mixed with atelocollagen to a rat femoral transverse fracture with the purpose of enhancing bone healing by stimulating osteogenesis, chondrogenesis, as well as angiogenesis [218]. Bone union at the fracture site with increased capillary density could be confirmed by radiographic, μCT and histological evaluation at 8 weeks after administration. Inhibition of miR-222 promoted osteogenic (RUNX2, COL1A1, OCN) or chondrogenic (COL2A1, aggrican, SOX9) differentiation in hMSCs, as determined by expression of osteogenic or chondrogenic markers, respectively. 

**Figure 2:**
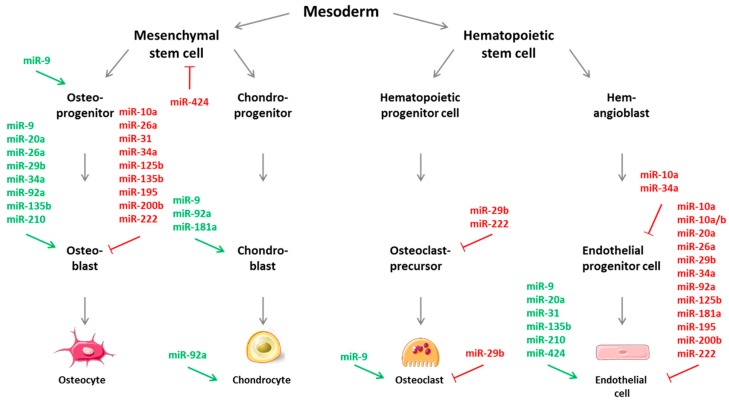
Regulating effects of microRNAs (miRs/miRNAs) involved in the regulation and coupling of bone angiogenesis (“CouplingmiRs”) during cell fate determination. MiRNAs were assigned with their individual positive/stimulatory (green colored) or negative/inhibitory (red colored) function and occurrence during the specific differentiation steps of osteogenic and angiogenic cells. Cell images from Servier Medical Art by Servier licensed under a Creative Commons Attribution 3.0.

In this review, miRNAs or “CouplingmiRs/CPLGmiRs” were identified that are known so far to regulate, either positively or negatively, angiogenesis as well as osteogenesis function—as potent molecular managers—that may simultaneously regulate multiple endogenous signaling cascades. This has been summarized in Figure 2, where the described miRNAs have also been depicted with regard to their allocation during the developmental fate of osteogenic or angiogenic cells. As for many miRNAs in general, also for coupling miRs/CPLGmiRs, most mechanisms of action are negative/inhibitory in nature for the regulation of osteogenesis as well as angiogenesis. Also noteworthy, but not surprising, is the finding that most miRNAs potentially involved in angiogenic-osteogenic coupling are encountered in the osteoblastic lineage rather than in chondroblasts or osteoclasts. And miRNAs are particularly present during the differentiation of osteoblasts, while they exert their modulatory effects during angiogenesis mostly in mature endothelial cells. Delivery of miRNAs may provide a way to maximally mimic the native bone development environment, and thus possess the therapeutic potential to enhance bone regeneration and repair. In contrast, miR inhibitors, i.e., antisense oligonucleotides directed against miRNAs, can be applied in the form of antagomiRs or LNA-antimiRs [58,179,219]. RNA/DNA hybrids modified by this locked nucleic acid-technology represent single stranded modified antisense oligonucleotides with increased stability and affinity and also facilitate cell penetration. Furthermore, they are biocompatible, as they cause no cellular immune response, are highly soluble, and have already been tested by various in vitro and in vivo studies as well as clinical trials (e.g., miR-122 LNA anti-miRNA oligonucleotides for Hepatitis C virus or miR-34 for solid cancers) [220,221]. Additionally, any new findings in this field of research might additionally yield considerable anti-angiogenic targets for tumor therapies, as tumor angiogenesis—i.e., the formation of tumor-associated angiogenic vessels—is a key requirement in tumor growth, progression, and metastasis. As an example, it would be very favorable to identify angiomiRs that negatively regulate VEGF expression in combination with current anti-VEGF therapies. Inversely, the identification of novel oncomiRs could also pave the way and lead to the description of unknown miRNAs with a function in bone angiogenesis [222,223,224]. Currently, microRNA-targeting therapy is still in development due to new challenges compared to conventional drugs, and results of experimental studies in animal models need to be transferred to clinical applications. Nevertheless, in addition to their role as potential angiogenic or bone regenerative therapeutic targets, miRNAs are emerging as distinguished disease biomarkers relevant to specific physiologic or pathologic conditions which could serve for immediate use in cardiovascular and bone diseases.

## Figures and Tables

**Table 1:** Summary of microRNAs with a presumed role in osteogenic-angiogenic coupling paving the way for bone angiogenesis (“CouplingmiRs/CPLGmiRs”).

MicroRNAs	Targets ^1^	Regulatory Role	Effects	Study Models	Ref.
MiR-9	VEGF, VE-CAD (CD144)	AMPK signaling pathway	Enhanced osteogenic diff. & mineral.; increased angiogenesis	MC3T3-E1	[106]
	DKK1	COL1, OCN, BSP; ALP activity	OB diff. & mineralization	C2C12 cells	[107]
	SOCS5	JAK-STAT signaling pathway	Promotion of EC migration & angiogenesis	Primary microvascular ECs, HUVECs	[108]
	Cbl	Bim ubiquitination, apoptosis	Promotion of OC survival	OC, OC precursor cells (RAW264.7)	[110]
MiR-10a	β-catenin, LEF1; VEGF, VE-CAD (CD144), cyclin D1, MMP2	Wnt signaling; angiogenesis-related gene expression	Inhibition of osteogenic diff. & blood vessel formation	MC3T3-E1 MUVECS	[111]
	HMGA2	β–galactosidase expr; p16Ink4a/p19Arf expression	EPC senescence & angiogenesis; self-renewal potential	lin−BM-MSCs	[113]
MiR-10a/10b	MIB1	Notch signaling	Regulating blood vessel outgrowth/tip cell behavior	HUVECs	[115]
MiR-20a	BMP2, BMP4, RUNX2	Effects BMP/RUNX2 signaling positively; blocks OB inhibitors & PPARγ	Enhances osteogenic differentiation; suppresses adipogenesis	hBM-MSC	[116]
	JAK1; p21, S1P receptor EDG	Downregulation of proangiogenic JAK 1 & cell cycle inhibitors	Inhibits EC sprout formation	HUVECs	[117]
	TNFSF15	VEGF-AKT/ERK –miR20a/31 signaling	Stimulation of angiogenesis	HUVECs	[118]
MiR-26a	VEGF, ANG1, RUNX2, BMP2 OCN, ALP; GSK3β	WNT signaling activation	Enhanced angiogenesis & bone regeneration	Primary hBM-MSC, MC3T3-E1	[76,98]
	VEGF	PIK3C2α/AKT/HIF-α/VEGFA pathway	Inhibition of angiogenesis;	HUVECs	[141]
	SMAD1	BMP signaling inhibition	OB differentiation	hADSCs	[119]
	SMAD1	BMP signaling	Inhibits EC growth, proliferation, migration; regulates early angiogenesis	HUVECs	[123]
MiR-29b	TGF-β3, HDAC4, ACTVR2A, CTNNBIP1, DUSP2; COL1A1, 5A3, 4A2	Silences neg. osteogenic regulators suppresses ECM protein synthesis	Promotes osteoblastogenesis at multiple stages	MC3T3 pre-OB	[124]
	c-FOS	Reduced TRAP expr., lacunae generation, collagen degradation	Neg. regulator of human OC differentiation and activity	OC (CD14 +)	[125]
	TNFSF15	TNFSF15-enhanced JNK-GATA3 signal. & VEGF inhibition	Suppression of VEGF secretion	Mouse EC line bEnd.3	[126]
	AKT3	Inhibition of tumor vascularization via VEGF & cancer cell activity via c-MYC	Anti-angiogenic and anti-tumorigenic role	HUVECs, Breast cancer cells	[127]
MiR-31	OSX	Downregulation of OSX	Influences osteogenic differentiation	hMSC; Osteosarcoma cell	[129]
	Satb2 protein	Inhibition by RUNX2; Upregulation of Satb2 protein & osteogenic TF	Induces BM-MSC osteogenic differentiation	hBM-MSC	[130]
	E-selectin	Regulation of E-selectin expression	Inhibition of angiostatin-induced angiogenesis; TNF-mediated induction of endothelial adhesion	HUVECs	[84]
	TNFSF15	VEGF-AKT/ERK –miR20a/31 signaling	Stimulation of angiogenesis	HUVECs	[118]
MiR-34a	Jagged1	Regulation of cell cycle regulator & proliferation proteins & Jagged1	Inhibition of osteoblast differentiation	hMSC; mouse heterotopic bone formation model	[132]
	JAGGED1	Activation of Notch signaling	Induction of glucocorticoid-mediated osteogenic differentiation	hMSC	[133]
	RBP2	Promotes mineral, ALP activity & RUNX2 expression; downreg. NOTCH1 & Cyclin D1 expr.	Promotion of osteogenic differentiation; enhanced heterotopic bone formation	hADSCs; mouse heterotopic bone formation model	[134]
	VEGF	Inhibitory effects of dexamethasone on EC viability & VEGF	Decreased blood vessel development	Rat Glucocorticoid- induced osteonecrosis	[137]
	SIRT1	Increased SIRT1 expr. & FOXO1 acetylation regulating vascular EC homeostasis	Inhibition of EPC-mediated angiogenesis	Rat EPC	[138]
	E2F3a, survivin	Interference with VEGF secretion, EC proliferation & migration	Dysregulated tumor angiogenesis	HNSCC tumors & cells	[140]
MiR-92a	?	?	Enhanced fracture healing & inhib. of neovascularization	Mice with femoral fracture	[142]
	HGF, ANGPT1	ITGA5, MEK4	Inhibition of tube formation by HUVECs	hADSCs	[143]
	?	integrin a5, sirtuin1, eNOS	Attenuates neointimal lesion by accelerating re-endothelialization	MiR-92a knockout mice	[144]
MiR-125b	OSX	RUNX2, a-SMC, ALP, matrix mineralization	Calcification of vascular smooth muscle cells	HCASMCs	[145]
	ErbB2	?	Inhibits OB diff by downreg. of cell proliferation	ST2 cells (mMSCs)	[146]
	VEGF, ERBB2		Regulation of angiogenesis during wound healing	HUVECS	[147]
	Cbf-beta	ALP, OCN, OPN	Inhibition of osteogenic differentiation	C3H10T1/2	[148]
	SMAD4	ALP, RUNX2	Downregulation of osteogenic differentiation	hMSCs	[149]
	VE-Cadherin		Inhibition of blood vessel (tube) formation	HUVECs	[150]
MiR-135b	?	?	OB differentiation	hBM-SCs	[151]
	HIF-1	?	Enhanced endothelial tube formation	Human MM cells; HUVECs	[152]
	SMAD5	?	Impaired osteogenic differentiation	hMSCs	[153]
MiR-181a	?	CCN1, aggrecan	Maintaining homeostasis of chondrocytes	Human HCS-2/8 cells	[154]
		COL10A1	Chondrocyte differentiation	hMSC	[155]
	RGS16	CXCR4 signaling; VEGF, MMP1	Angiogenesis & metastasis in chondrosarcoma	Xenograft mice; JJ chondrosarc. cells	[156]
	?	VEGF expression	Chondrosarcoma-associated angiogenesis	JJ chondrosarc. cell line	[157]
	Cbl	Bim ubiquitination, apoptosis	promote OC survival	OC, OC precursor cells (RAW264.7)	[110]
MiR-195	?	VEGF	Osteogenic diff. & proliferation; control of angiogenesis	hMSC(MC3T3) chick chorio-allantoic membrane	[158]
	?	VEGF, VAV2 CDC42	HCC-associated angiogenesis & metastasis; migration & capillary tube form. of ECs	QGY-7703, MHCC-97H HCC cells; HUVECs	[159]
MiR-200b	ZEB1	ZEB1-TF target genes	Inhibits proliferation, migration & invasion of osteosarcoma cells	OsteosarcomaU2OS, Saos2, HOS, MG63	[160]
	VEGF-A; ZEB2, ETS1, KDR,GATA2	Decreases VEGF-A expression & TF-target genes	Inhibition of VEGF-A induced osteogenesis; Inhibition of TF-activated angiogenesis	Rat BM-MSC & HUVEC coculture	[161]
	VEGF, FLT-1, and KDR	VEGF-induced phosph. of ERK1/2	Inhibition of angiogenesis; red. capillary formation	A549 cells, HUVECs	[162]
MiR-210	AcvR1b	Inhib. of TGFb/activin signaling	Promotes OB differentiation	ST2 stromal cells	[163]
	VEGF	PPARgamma, ALP, OSX	Promoteion of OB diff., inhibition of adipocyte diff.	hBM-SCs, 17β-estradiol (E2)treated OB	[164]
	EFNA3	VEGF-expression mediated angiogenesis	EC survival, diff., migration; stim. of tubulogen. & chemotaxis	HUVECs	[165]
MiR-222	SMAD 1, 5, 8 protein & phosphoryl.	Decreased SMAD5-RUNX2 signaling & OSX, ALP, and OC levels & mineral.	Neg. regulator of osteogenic differentiation	hBM-SC	[166]
	c-Src, Dcstamp	RANKL-induced expression of TRAP & cathepsin K	Inhibitory regulator of c-Src-mediated osteoclastogenesis	RAW264.7 pre-OC cells	[167]
	c-KIT	Suppression of tube formation, wound healing, cell migration via SCF	Inhibitory regulation of in vitro angiogenesis	HUVEC	[168]
MiR-424	RUNX, CBFβ, BMP	Osteogenic diff. of hMSCs	Bone formation	hMSCs	[169]
	MAPK, WNT & insulin signal.	OB differentiation of hMSCs	Bone formation	hMSCs	[170]
	FGF-2; via FOXO1	Decrease of ALP, mineralization & osteog. markers	Enhances proliferation & osteogenic differentiation of hMSCs	Pigs, cellular oxidative stress model	[171]
	CUL2; via RUNX-1→ C/EBPα→ PU.1	Stabilization of HIF-1α	Regulation of Angiogenesis	ECs, ischemic tissues	[101]

^1^ Identified target genes or downstream effectors; HUVECs, human umbilical vein endothelial cells; ?, unknown molecular target(s) and/or regulatory role; MUVECs, mouse umbilical vein endothelial cells; EC, endothelial cells; EPC, endothelial progenitor cells; hMSCs, human mesenchymal stem cells; hBM-SC, bone-marrow derived stem cells; hADSCs, human adipose-derived mesenchymal stem cells; TNFSF15; cytokine tumor necrosis factor superfamily 15; OB, osteoblasts; OC, osteoclasts; TF, transcription factor.
